# Prognostic Impact of Internal Jugular Vein Invasion Through Extrathyroidal and Extranodal Extension in Papillary Thyroid Carcinoma

**DOI:** 10.1002/wjs.70366

**Published:** 2026-04-09

**Authors:** Ai Matsui, Yoshiyuki Saito, Kosuke Inoue, Kenichi Matsuzu, Wataru Kitagawa, Kiminori Sugino, Koichi Ito

**Affiliations:** ^1^ Department of Surgery Ito Hospital Tokyo Japan; ^2^ Department of Social Epidemiology Graduate School of Medicine and School of Public Health Kyoto University Kyoto Japan

**Keywords:** extranodal extension, extrathyroidal extension, internal jugular vein, papillary thyroid carcinoma

## Abstract

**Background:**

IJV invasion is a rare but significant occurrence in PTC. Although the current staging system categorizes *T* stages based on organ invasion, the classification for IJV invasion remains unclear. We evaluated the prognostic impact of internal jugular vein (IJV) involvement by extrathyroidal extension (ETE) and extranodal extension (ENE) in papillary thyroid carcinoma (PTC) and investigated the appropriate T‐stage classification for ETE to the IJV.

**Methods:**

This retrospective study included PTC patients who underwent surgery between 2005 and 2011 at our hospital. We analyzed patients with IJV resection due to ETE or ENE, dividing them into ETE and ENE groups. We also compared the ETE group's prognoses with those of patients with stage III or IV PTC to evaluate the T4a vs. T4b classification.

**Results:**

Among 5482 PTC cases, 17 were in the ETE group and 47 in the ENE group. We compared the ETE group's prognoses with those of the patients with stage III or IV PTC to evaluate the T4a vs. T4b classification. Compared to the ENE patients, the ETE patients had significantly lower 10‐year overall survival (40.6% vs. 81.9%; HR 2.77, 95% CI: 0.99–7.67) and disease‐specific survival (44.3% vs. 92.6%; HR 6.81, 95% CI: 1.68–27.51). Survival for ETE to the IJV was significantly worse than for stage III PTC but comparable to stage IV.

**Conclusions:**

ETE to the IJV showed a poor prognosis, comparable to stage IV, whereas ENE to the IJV had a favorable prognosis even though we did not exclude the presence of high‐risk factors. ETE to the IJV represents an aggressive disease course in PTC, warranting careful consideration in staging and treatment planning.

## Graphical abstract

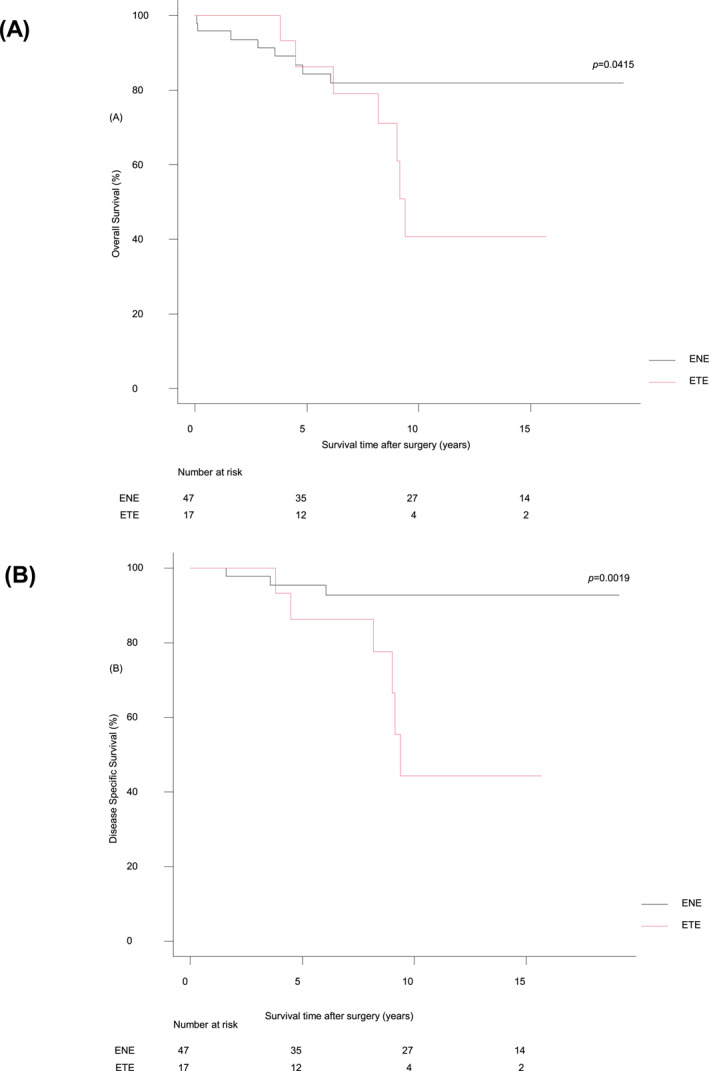

## 1 Introduction

Papillary thyroid carcinoma (PTC) is the most common type of cancer in the thyroid and is typically a slow‐growing tumor. Active surveillance is thus considered an acceptable management option for small PTC lesions [1, 2]. Even in cases of lymph node metastases, the prognosis remains favorable, prompting discussions about the feasibility of less‐extensive surgical approaches [3, 4, 5]. However, cases involving extrathyroidal extension (ETE) may exhibit a more aggressive clinical course.

In the 8th edition staging system by the American Joint Committee on Cancer/Union for International Cancer Control (AJCC/UICC) [6], neither the location of lymph node metastases (i.e., N1a vs. N1b) nor the presence of extranodal extension (ENE) directly influences the disease stage. In contrast, that system classifies ETE based on the specific organs involved, resulting in its designation as T3b, T4a, or T4b. If gross ETE extends to the strap muscles, it is classified as T3b. If the ETE involves subcutaneous soft tissues, the larynx, trachea, esophagus, or recurrent laryngeal nerve, it is classified as T4a. Cases in which ETE affects the prevertebral fascia or encases the carotid artery or mediastinal vessels are classified as T4b. The specific organs involved significantly influence the disease stage, with affected cases being assigned to stage II, III, or IV in patients aged ≥ 55 years, depending on the organs invaded by the primary tumor.

These classifications can generally be applied without issue when using the 8th edition of the AJCC/UICC staging system. However, there are instances in which the primary tumor may involve the invasion of one or more organs that are not specifically defined within the AJCC/UICC system for T‐classification. These undefined areas can present challenges when cases are registered in databases such as cancer registries. To address this, the U.S. National Cancer Institute's Surveillance, Epidemiology, and End Results (SEER) Program Registrar Staging Assistant [7] provides criteria concerning how to assign appropriate T‐classifications for ETE involving organs not covered by the AJCC/UICC system. Similarly, the Japanese General Rules for the Description of Thyroid Cancer, 9th Edition (referred to hereafter as the ‘Japanese description’) [8] provides comparable criteria for staging such cases.

The internal jugular vein (IJV) is one of the organs not referenced in the AJCC/UICC staging system. ETE to the IJV corresponds to T4b and is classified as stage IV for patients aged ≥ 55 years in the SEER system, and it corresponds to T4a and is classified as stage III for the same age group in the Japanese description. Two studies indicated that the IJV is the most commonly invaded organ in ENE within the lateral neck area [9, 10], and thus invasion of the IJV has been observed in both ETE and ENE. For the AJCC/UICC, there are no classification rules for ENE, and the N‐classification is determined by lymph node levels rather than the presence of ENE.

We conducted the present study to evaluate the difference in prognosis between ETE and ENE when the IJV is involved. We also compared the prognoses of patients with ETE to the IJV with stage III or stage IV PTC in order to determine which classification (T4a or T4b) more accurately correlates with staging outcomes.

## 2 Study Design and Patients

### 2.1 Patients

We selected the cases of patients who underwent surgery for PTC at our hospital in Tokyo during the period from 2005 to 2011. Of these patients, we selected those whose IJV was excised because of ETE or ENE invasion. We defined ‘combined resection of the IJV’ based on intraoperative findings, but we excluded the cases of patients who underwent a combined resection of the IJV due to vascular damage. Cases involving adhesion of the IJV were not included. We also excluded cases with another cancer or containing components of another malignancy. We defined the enrolled patients as belonging to two groups: the ETE group and the ENE group.

This study also focused on the differences in the stage classification between the SEER and the Japanese description. ETE to the IJV corresponds to T4b and is classified as stage IV for patients ≥ 55 years old in SEER, and it corresponds to T4a and is classified as stage III for the same age group in the Japanese description. We thus selected clinical stage III and stage IV cases to compare the prognosis of ETE to the IJV with stage III and stage IV cases. The research period for the stage III and IV cases was the same as that for ETE and ENE. Approval to conduct this clinical study was granted by the hospital's Institutional Review Board. This study was retrospective in nature, and the requirement for informed consent was waived by the Ito Hospital Institutional Review Board. Instead, an opt‐out approach was used for patient participation.

### 2.2 Follow‐Up and Recurrence Cases

Most of the patients were surveyed after their surgery as described [11, 12]. In short, postoperative visits were scheduled every 6 months. A neck ultrasound and blood tests were performed at each visit. Computed tomography (CT) examinations of the chest were performed every 5 years postoperatively and/or prior to recurrence surgery. An additional CT examination was performed for the patients diagnosed with lymph node recurrence by ultrasound or suspected of recurrence based on changes in their serum thyroglobulin levels. However, the examination of advanced cases (e.g., cases of incomplete tumor resection or distant metastases) was conducted on a case‐by‐case basis. Distant metastases are typically defined as metastases identified by a CT examination or radioactive iodine (RAI) therapy after surgery. In some cases, PET‐CT is also used. Regional recurrence was defined as the diagnosis of PTC via fine‐needle aspiration.

### 2.3 Statistical Analyses

The study's primary outcome was overall survival (OS). A survival analysis was performed using the Kaplan‐Meier method, and a Cox proportional hazards regression analysis was conducted. Probability (*p*)‐values < 0.05 were considered significant. All statistical analyses were performed using EZR (Saitama Medical Center, Jichi Medical University, Saitama, Japan), which is a graphical user interface for R (The R Foundation for Statistical Computing, Vienna, Austria). More precisely, it is a modified version of R Commander (ver. 2.8–0) designed to include statistical functions that are frequently used in biostatistics [13]. Fisher's exact test was used for the comparison of categorical variables. Student's test was used to compare continuous variables. The OS and disease‐specific survival (DSS) rates were estimated using the Kaplan‐Meier method and compared using stratified log‐rank tests.

## 3 Results

### 3.1 Patient Characteristics

The total number of PTC cases at our hospital during the study period was 5482. Among these, 64 cases (1.2%) involved IJV excisions. Of these, 17 cases (0.3%) had ETE to the IJV, and 47 cases (0.9%) had ENE to the IJV. Excluding cases of ETE to the IJV, the number of stage III cases was 139, and that of stage IV cases was 80. The patients' backgrounds in ETE and ENE cases are summarized in Table 1. Significant between‐group differences were observed in age and tumor size. The median age was significantly higher in the ETE group compared to the ENE group: median (interquartile range [IQR]) 72 years (68, 75) vs. 65 (53.5, 71) years, respectively (*p* = 0.042). In addition, the tumor size was significantly larger in the ETE group compared to the ENE group: 41.9 mm (31.6, 61.0) vs. 20.5 mm (15.3, 26.2), respectively (*p* < 0.001). Detailed analyses of additional clinicopathological characteristics in cases with IJV resection are provided in the Supporting Information S1.

**TABLE 1:** Characteristics of 64 patients with papillary thyroid carcinoma and either extrathyroidal extension (ETE) or extranodal extension (ENE) to the internal jugular vein.

	ETE (*n* = 17)	ENE (*n* = 47)	*p*‐value
Age, yrs	72 (68, 75)	65 (53.5, 71)	**0.042**
Sex			0.35
Male	3 (17.6)	16 (34)	
Female	14 (82.4)	31 (66)	
Tumor size, mm	41.9 (31.6, 61.0)	20.5 (15.3, 26.2)	**<** **0.001**
Lymph node size, mm	—	22.7 (15.4, 28.3)	
Distant metastasis	4 (23.5)	7 (14.9)	0.45
Extent of thyroidectomy			0.091
Total thyroidectomy	10 (58.8)	39 (83.0)	
Lobectomy	7 (41.2)	8 (17.0)	
Central neck dissection	17 (100)	47 (100)	1.000
Lateral neck dissection	16 (94.1)	47 (100)	0.27
Incomplete tumor resection (%)	4 (23.5)	11 (23.4)	1.000
Radioactive iodine therapy	5 (29.4)	14 (29.8)	1.000
External‐beam radiotherapy	1 (5.9)	1 (2.1)	0.46

*Note:* Data are number (%) or median (IQR). Bold values indicate statistical significance (*p* < 0.05).

Abbreviations: ENE: extranodal extension to the internal jugular vein (IJV), ETE: extrathyroidal extension to IJV.

In the ETE group, five patients underwent radioactive iodine (RAI) therapy, and one patient received external radiotherapy after surgery. In the ENE group, 14 patients underwent RAI therapy, and one patient received external radiotherapy after surgery.

### 3.2 The 10‐Year OS and DSS Rates in the ETE and ENE Groups

**FIGURE 1:**
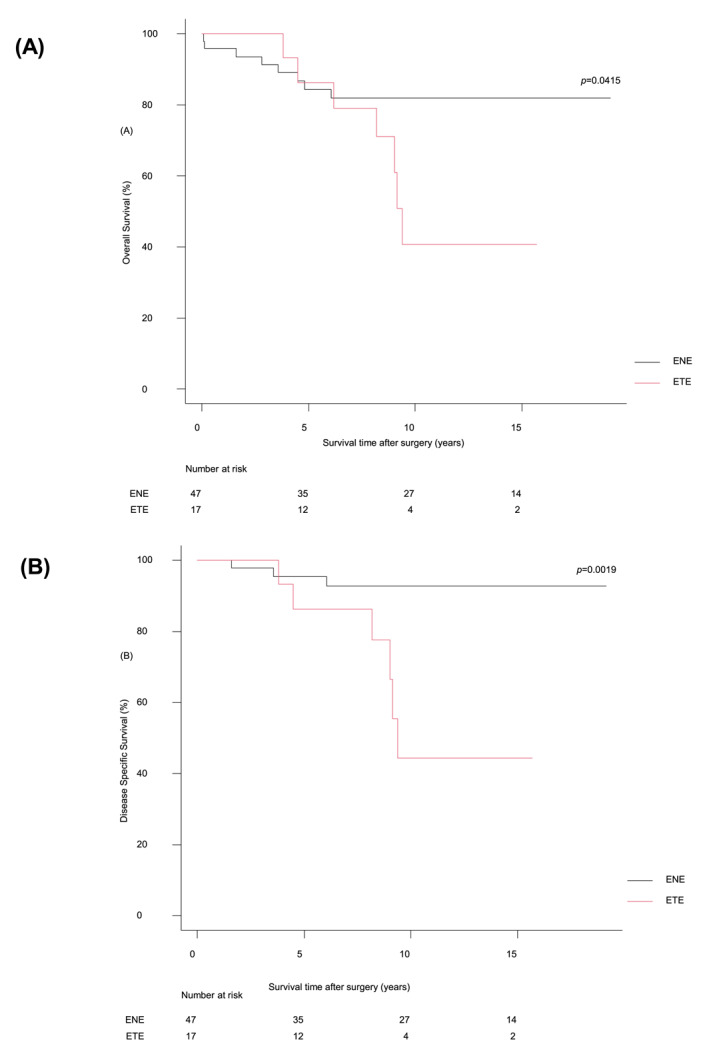
(A) Kaplan‐Meier survival curves comparing the overall survival (OS) between the cases of the patients with extrathyroidal extension (ETE) and those with extranodal extension (ENE) to the internal jugular vein (IJV). The 10‐year OS rates were 40.6% and 81.9%, respectively. (B) Kaplan‐Meier survival curves comparing the disease‐specific survival (DSS) between the ETE and ENE groups. The 10‐year DSS rates were 44.3% and 92.6%, respectively.

Figure 1 depicts the Kaplan‐Meier estimates of OS and DSS for the ETE and ENE groups. The median follow‐up duration was 13.8 years. The 10‐year OS and DSS rates were significantly lower for the ETE group compared to the ENE group. The 10‐year OS rate was 40.6% for ETE and 81.9% for ENE: hazard ratio (HR) 2.77, 95% confidence interval (CI): 0.99–7.67. The 10‐year DSS rate was 44.3% for ETE and 92.6% for ENE (HR 6.81, 95% CI: 1.68–27.51). Detailed analyses of patterns of recurrence and causes of death in cases requiring IJV resection are provided in the Supporting Information S1.

### 3.3 ETE to the IJV and the T‐Classification in the U.S. and Japan

Table 2 describes the invasion of organs and clinical T‐classification according to the AJCC/UICC, SEER, and the Japanese descriptions for the cases of ETE to the IJV. There were 10 patients (58.8%) with ETE to the IJV and other organs (e.g., the esophagus, trachea, or carotid artery) simultaneously. According to the Japanese descriptions, these patients' cases were staged as T4a with the exception of those with invasion of the carotid artery (two cases, 11.8%). In SEER, cases involving invasion of the IJV are staged as T4b.

**TABLE 2:** The cases of extrathyroidal extension (ETE) invading the internal jugular vein (*n* = 17): Invasion of other organs by the tumor, and clinical T‐classification by the AJCC/UICC, SEER, and the Japanese description.

Organ	Clinical T‐classification			Japanese description
	Cases (%)	AJCC/UICC	SEER	
Internal jugular vein	17 (100)	Undefined	T4b	T4a
Esophagus	2 (11.8)	T4a	T4a	T4a
Larynx	1 (5.9)	T4a	T4a	T4a
Recurrent laryngeal nerve	3 (17.6)	T4a	T4a	T4a
Vagus nerve	1 (5.9)	Undefined	T4a	Undefined
Sternocleidomastoid muscle	3 (17.6)	Undefined	T4a	T4a
Trachea	2 (11.8)	T4a	T4a	T4a
Thyroid cartilage	1 (5.9)	Undefined	T4b	T4a
Carotid artery	2 (11.8)	T4b	T4b	T4b

*Note:* AJCC/UICC, The American Joint Committee on Cancer/Union for International Cancer Control. SEER: The National Cancer Institute's Surveillance, Epidemiology, and End Results. Japanese description: General Rules for the Description of Thyroid Cancer 9th edition by the Japanese Society of Thyroid Pathology and Japan Association of Endocrine Surgery. SEER provides cancer statistics in the United States, while the Japanese description is used for cancer management and registration in the National Clinical Database in Japan. To clarify the differences in *T* staging, we have outlined the T‐classification for the IJV and other organs based on the AJCC/UICC, SEER, and the Japanese description.

Table 3 shows the differences in staging for the cases of ETE to the IJV based on SEER and the Japanese descriptions. Most of the ETE cases occurred in patients aged ≥ 55 years (*n* = 16, 94.1%). Patients aged ≥ 55 years old with ETE to the IJV are classified as T4b and stage IV in the U.S., whereas in Japan they are classified as T4a and stage III. According to the SEER classification, the present stage distribution (I–IV) was 5.9% for stage I (*n* = 1), 0% for stage II (*n* = 0), 0% for stage III (*n* = 0), and 94.1% for stage IV (*n* = 16). With the Japanese description, the corresponding results were 5.9% (*n* = 1), 0% (*n* = 0), 64.7% (*n* = 11), and 29.4% (*n* = 5), respectively. Among the five cases classified as stage IV according to the Japanese description, four cases had distant metastases, and one case involved invasion of the carotid artery without distant metastases.

**TABLE 3:** Staging in the United States and Japan: extrathyroidal extension (ETE) to the internal jugular vein (*n* = 17).

Stage	SEER	Japanese description
I	1 (5.9)	1 (5.9)
II	0 (0)	0 (0)
III	0 (0)	11 (64.7)
IV	16 (94.1)	5 (29.4)

*Note:* The data are *n* (%). ETE to the IJV corresponds to T4b and is classified as stage IV for patients aged ≥ 55 years in SEER, while it corresponds to T4a and is classified as stage III for the same age group in the Japanese description.

**FIGURE 2:**
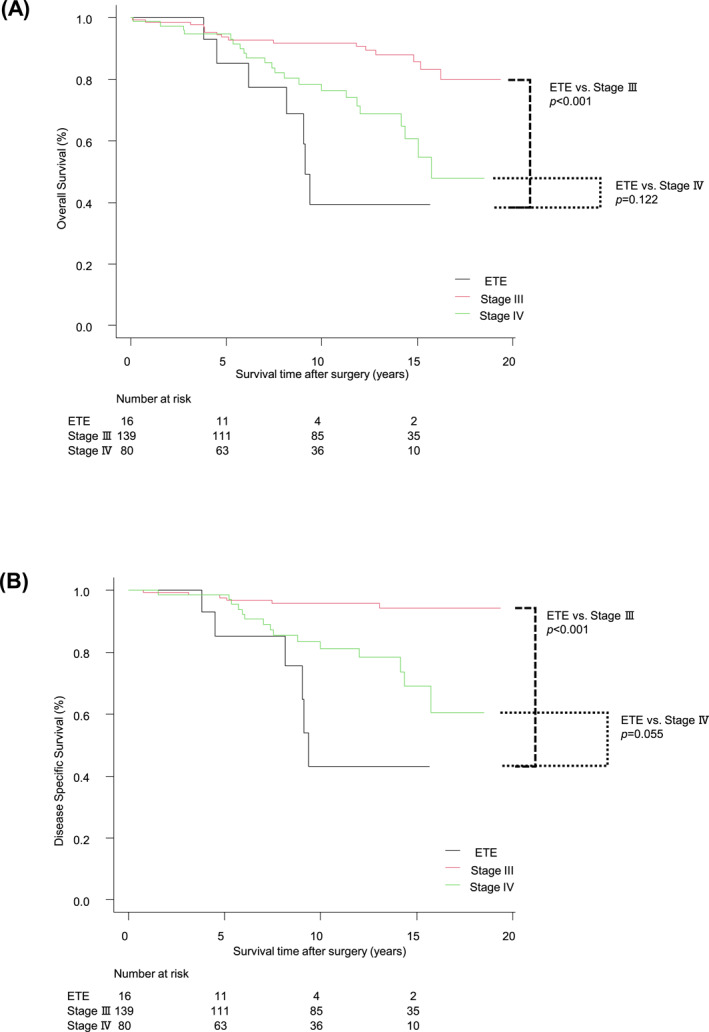
(A) Kaplan‐Meier survival curves comparing the OS among patients aged ≥ 55 years with ETE to the IJV, stage III, and stage IV. The 10‐year OS rates were 39.3%, 91.8%, and 76.3%, respectively. (B) Kaplan‐Meier survival curves comparing the DSS among patients aged ≥ 55 with ETE to the IJV, stage III, and stage IV. The 10‐year DSS rates were 43.2%, 95.7%, and 81.2%, respectively.

To determine the appropriate staging, we compared the survival rates between the PTC cases with ETE to the IJV in patients aged ≥ 55 and those of stage III or IV (Figure 2). The cases with ETE to the IJV showed significantly worse 10‐year OS and DSS compared to the stage III cases (OS: 39.3% vs. 91.8%, HR 0.43, 95% CI: 0.27–0.67; DSS: 43.2% vs. 95.7%, HR 0.30, 95% CI: 0.17–0.53). However, no significant differences were observed between the ETE cases and the stage IV cases (OS: 39.3% vs. 76.3%, HR 0.80, 95% CI: 0.60–1.07; DSS: 43.2% vs. 81.2%, HR 0.73, 95% CI: 0.54–1.02).

**FIGURE 3:**
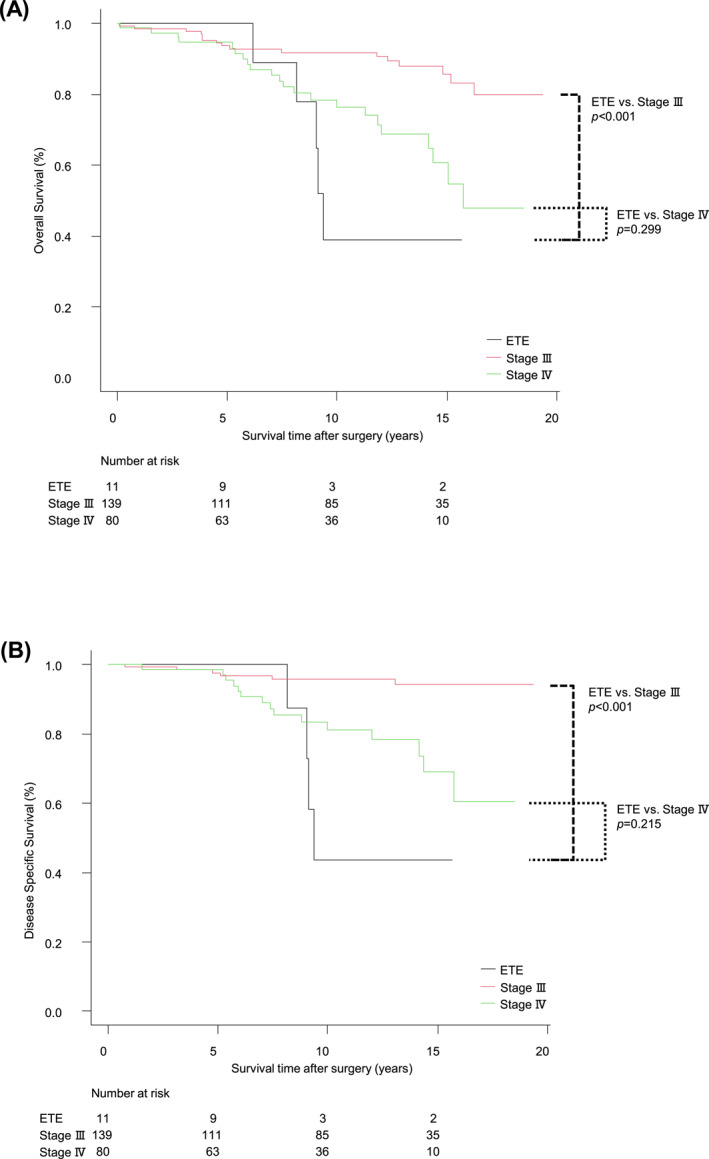
(A) Kaplan‐Meier survival curves comparing the 10‐year OS among patients aged ≥ 55 ETE to the IJV without M1 or other T4b factors, stage III, and stage IV. The 10‐year OS rates were 38.9%, 91.8%, and 76.3%, respectively. (B) Kaplan‐Meier survival curves comparing the 10‐year DSS among the cases of ETE to the IJV without M1 or other T4b factors, stage III, and stage IV. The 10‐year DSS rates were 43.8%, 95.7%, and 81.2%, respectively.

As shown in Tables 1 and 2, the patients with ETE to the IJV had additional factors leading to stage IV classification, such as M1 and other T4b factors (i.e., carotid artery and thyroid cartilage). To clarify the impact of ETE to the IJV itself, we performed an analysis excluding cases with other stage IV factors, limiting the comparison to 11 patients aged ≥ 55 with ETE to the IJV alone (Figure 3). The results showed a similar trend: ETE to the IJV remained associated with significantly worse 10‐year OS and DSS compared to stage III (OS: 38.9% vs. 91.8%, HR 0.45, 95% CI: 0.27–0.75; DSS: 43.8% vs. 95.7%, HR 0.32, 95% CI: 0.17–0.61). However, no significant differences were observed compared to stage IV (OS: 38.9% vs. 76.3%, HR 0.84, 95% CI: 0.61–1.17; DSS: 43.8% vs. 81.2%, HR 0.79, 95% CI: 0.55–1.15).

## 4 Discussion

Our analyses of 64 cases of PTC revealed that ETE to the IJV resulted in significantly poorer survival rates compared to ENE to the IJV. Both ETE and ENE may serve as prognostic factors, but although both involve the IJV, their clinical behavior differs significantly. Consequently, their management, including treatment strategies and follow‐up approaches, may also need to be distinct. The T‐classification for ETE to the IJV is not explicitly defined in the AJCC/UICC staging system. In Japan, it is classified as T4a, whereas in the U.S., it is classified as T4b. As a result, patients aged ≥ 55 years with ETE to the IJV are classified as having stage III disease in Japan and stage IV in the U.S. In the present series of patients, the survival rate for ETE to the IJV was comparable to that of stage IV. These findings suggest that classifying ETE to the IJV as T4b may be more appropriate.

In the AJCC/UICC staging system, the T‐classification of ETE cases varies based on the invaded organs. For example, invasion of the strap muscles is classified as T3b, the trachea or recurrent laryngeal nerves as T4a, and the carotid artery or mediastinal vessels as T4b. This system does not explicitly address the IJV invasion. The SEER and Japanese classifications designate IJV invasion as T4b and T4a, respectively (Table 2). This discrepancy affects the staging of patients aged ≥ 55 years [7, 8]. With the exception of one case involving a patient < 55 years old in our series, all of the patients with ETE to the IJV were aged ≥ 55 years. For patients with ETE to the IJV, the distinction between T4a and T4b significantly influences their staging and is a critical clinical consideration. Determining whether ETE to the IJV is classified as T4a or T4b holds substantial importance in guiding appropriate treatment strategies and follow‐up planning.

There have been case reports about the invasion of the IJV by thyroid cancer, suggesting that such invasion indicates a poor outcome [14, 15], but our search of the relevant literature identified no comprehensive studies specifically focused on ETE to the IJV. In our series, all cases represented direct invasion of the IJV by PTC, and no tumor thrombus was observed. A recent systematic review found no co‐existing direct vessel wall invasion in cases of IJV tumor thrombus, suggesting a mechanism distinct from direct venous invasion [16]. These entities should be considered distinct. Our present investigation provides valuable data on this underexplored area, contributing to a better understanding of the clinical and prognostic implications of ETE to the IJV and potentially aiding in the refinement of TNM staging for such aggressive thyroid tumors [17].

Approximately 60% of the present patients with ETE to the IJV also had simultaneous invasion of other organs. However, except for two cases involving carotid artery invasion, most of the cases remained classified as T4a under the Japanese description criteria and were therefore categorized as stage III. If ETE to the IJV were instead classified as T4b, these cases would be upstaged accordingly. Notably, the 10‐year OS and DSS of patients with ETE to the IJV were significantly worse than those of stage III and were comparable to those of stage IV. These findings support consideration of reclassifying ETE to the IJV as T4b.

The cases of our patients with ENE to the IJV showed better 10‐year OS and DSS compared to the cases of ETE to the IJV. In the American Thyroid Association (ATA) guidelines, gross ETE corresponds to a high risk, and ENE is not considered a high‐risk factor [18]. The impact of ENE on prognosis and recurrence remains controversial, with conflicting findings in the literature. However, ENE appears to be linked to recurrence to some extent, as clinical N1 cases with ENE have been reported to have an approx. 15%–32% locoregional recurrence rate [19]. Moritani et al. analyzed 60 cases of ENE and concluded that ENE did not affect the survival of patients with PTC [9]. However, their data also indicated that although the locoregional recurrence rates did not significantly differ between their patients with and without ENE, the distant recurrence rate was higher in those with invasive ENE. In our study, approx. 15% of the ENE cases were diagnosed with M1 disease, suggesting a tendency for ENE to coexist with distant metastases.

Other studies have also supported the association between ENE and distant metastases [20, 21]. In contrast, Wu et al. reported that ENE negatively impacted the prognosis and that DSS differed significantly between patients with and without ENE (10‐year DSS: 99% vs. 73%) [22]. In their study, the patients with ENE tended to have significantly larger lymph nodes and a higher frequency of T4 cases, suggesting a strong association with the high‐risk factors described by the ATA. In our study, the cases with ENE to the IJV also included M1 and R2 cases, indicating that the presence of ENE may be related to more advanced disease. The impact of ENE alone (in the absence of other high‐risk factors) thus remains unclear. Our analyses also revealed that the 10‐year DSS rate for ENE to the IJV was 81.9%, which seems more favorable than the 10‐year DSS of 73% reported by Wu et al. for ENE without distinguishing the invaded organs. Although a direct comparison is difficult, this finding suggests that DSS may vary depending on the invaded organs of ETE and the presence of other high‐risk factors.

### 4.1 Study Limitations

Several study limitations should be considered. We focused on PTC and did not consider follicular thyroid carcinoma (FTC), another type of differentiated thyroid cancer. Since the staging system does not distinguish between PTC and FTC, the recommendations of this study regarding the T‐classification for PTC may not be applicable to FTC. The number of cases involving invasion of the IJV was relatively small, making it difficult to draw definitive conclusions. RAI administration was not standardized across patients, which may have affected survival outcomes. Detailed subclassification according to the sEx system (sEx2a, sEx2b, and sEx3) could not be performed, as the study period preceded the introduction of this classification in the Japanese description (9th Edition), and operative records from that era did not consistently allow reliable retrospective differentiation. The prognosis of patients with ENE to the IJV may be more favorable when high‐risk factors are excluded. Moreover, as this study specifically focused on the impact of ETE and ENE on the invasion of the IJV, further research is needed to evaluate the impact of ENE alone. While the data used in this study were collected more than 10 years ago, this approach can better reflect the true outcomes of this slow‐growing cancer.

## 5 Conclusion

ETE to the IJV showed a poor prognosis, comparable to stage IV, whereas ENE to the IJV had a favorable prognosis. The AJCC staging system does not currently define the *T* stage for IJV invasion, but the results of this study suggest that ETE to the IJV should be considered equivalent to T4b, which could be valuable for future revisions of the TNM classification. Both favorable and unfavorable prognoses have been reported for ENE, but our analyses revealed that ENE to the IJV showed a favorable prognosis, even though we did not exclude the presence of high‐risk factors as defined by the ATA. Among patients with ETE, the prognosis varies based on the invaded organ(s), and stage classifications are adjusted accordingly. Similarly, the prognosis for ENE may depend on the invaded organ, and IJV involvement may represent a relatively favorable organ among cases with ENE. Further research is needed to confirm these findings.

## Author Contributions

**Ai Matsui:** writing – original draft, formal analysis, data curation. **Yoshiyuki Saito:** conceptualization, methodology, writing – original draft, project administration. **Kosuke Inoue:** methodology, formal analysis, writing – review and editing. **Kenichi Matsuzu:** conceptualization, writing – review and editing, validation. **Wataru Kitagawa:** writing – review and editing, validation. **Kiminori Sugino:** writing – review and editing, validation. **Koichi Ito:** supervision, writing – review and editing, validation.

## Funding

The authors have nothing to report.

## Conflicts of Interest

The authors declare no conflicts of interest.

## Supporting information


Supporting Information S1

## Data Availability

The data that support the findings of this study are available from the corresponding author upon reasonable request.

## References

[1] I. Sugitani , Y. Ito , A. Miyauchi , T. Imai , and S. Suzuki , “Active Surveillance Versus Immediate Surgery: Questionnaire Survey on the Current Treatment Strategy for Adult Patients With Low‐Risk Papillary Thyroid Microcarcinoma in Japan,” Thyroid 29, no. 11 (2019): 1563–1571, 10.1089/thy.2019.0211. 31441377 PMC6862943

[2] Y. Saito , K. Matsuzu , H. Takami , et al., “Active Surveillance vs. Surgery in Low‐Risk Papillary Thyroid Microcarcinoma Patients and the Risk of Loss to Follow‐Up,” Cancer Medicine 13, no. 16 (2024): e70123, 10.1002/cam4.70123. 39194351 PMC11350832

[3] I. Sugitani , H. Kazusaka , A. Ebina , W. Shimbashi , K. Toda , and K. Takeuchi , “Long‐Term Outcomes After Lobectomy for Patients With High‐Risk Papillary Thyroid Carcinoma,” World Journal of Surgery 47, no. 2 (2023): 382–391, 10.1007/s00268-022-06705-8. 35972533

[4] S. Xu , H. Huang , Y. Huang , et al., “Comparison of Lobectomy vs Total Thyroidectomy for Intermediate‐Risk Papillary Thyroid Carcinoma With Lymph Node Metastasis,” JAMA Surgery 158, no. 1 (2023): 73–79, 10.1001/jamasurg.2022.5781. 36449303 PMC9713681

[5] Y. Saito , K. Matsuzu , A. A. H. Abdelhamid , et al., “Lobectomy vs Total Thyroidectomy With Ipsilateral Lateral Neck Dissection for N1b Intermediate‐Risk Papillary Thyroid Carcinoma,” JAMA Otolaryngology – Head & Neck Surgery (2024), 10.1001/jamaoto.2024.3860. PMC11826362 39602155

[6] S. Edition , S. Edge , and D. Byrd , AJCC Cancer Staging Manual (AJCC cancer staging manual) (2017).

[7] NATIONAL CANCER INSTITUTE Surveillance , Epidemiology, and End Results Program.

[8] H. Kamma , Y. Ito , S. Suzuki , et al., “Japanese General Rules for the Description of Thyroid Cancer (9Th Edition) Established by the Japan Association of Endocrine Surgery and the Japanese Society of Thyroid Pathology,” Thyroid Science 2, no. 1 (2025): 100021, 10.1016/j.thscie.2024.100021.

[9] S. Moritani , “Impact of Invasive Extranodal Extension on the Prognosis of Patients With Papillary Thyroid Carcinoma,” Thyroid 24, no. 12 (2014): 1779–1783, 10.1089/thy.2014.0167. 25157399

[10] T. H. Zhou , B. Lin , F. Wu , et al., “Extranodal Extension is an Independent Prognostic Factor in Papillary Thyroid Cancer: A Propensity Score Matching Analysis,” Frontiers in Endocrinology 12 (2021): 759049, 10.3389/fendo.2021.759049. 34803921 PMC8595930

[11] K. Matsuzu , K. Sugino , K. Masudo , et al., “Thyroid Lobectomy for Papillary Thyroid Cancer: Long‐Term Follow‐Up Study of 1,088 Cases,” World Journal of Surgery 38, no. 1 (2014): 68–79, 10.1007/s00268-013-2224-1. 24081532

[12] Y. Saito , K. Matsuzu , K. Sugino , et al., “The Impact of Completion Thyroidectomy Followed by Radioactive Iodine Ablation for Patients With Lymph Node Recurrence of Papillary Thyroid Carcinoma,” Surgery 166, no. 3 (2019): 342–348, 10.1016/j.surg.2019.04.009. 31128851

[13] Y. Kanda , “Investigation of the Freely Available Easy‐to‐Use Software 'EZR' for Medical Statistics,” Bone Marrow Transplantation 48, no. 3 (2013): 452–458, 10.1038/bmt.2012.244. 23208313 PMC3590441

[14] E. Kebebew and O. H. Clark , “Locally Advanced Differentiated Thyroid Cancer,” Surgical Oncology 12, no. 2 (2003): 91–99, 10.1016/s0960-7404(03)00032-x. 12946480

[15] A. S. Dikici , O. Yıldırım , M. E. Er , et al., “A Rare Complication of the Thyroid Malignancies: Jugular Vein Invasion,” Polish Journal of Radiology 80 (2015): 360–363, 10.12659/pjr.894057. 26236418 PMC4509426

[16] B. J. Laurin , R. Ballard , I. Malik , and J. Mitchell , “Internal Jugular Vein Tumor Thrombus in Papillary Thyroid Cancer: Our Institution's Experience and a Systematic Review of the Literature,” Frontiers in Endocrinology 16 (2025): 1514455, 10.3389/fendo.2025.1514455. 40070593 PMC11893390

[17] P. Y. Marcy , J. Thariat , C. Chevenet , and A. Lacout , “Jugular Vein Invasion Diagnosis and Prognosis in Thyroid Carcinomas,” Polish Journal of Radiology 81 (2016): 268–269, 10.12659/pjr.896757. 27354880 PMC4907401

[18] B. R. Haugen , E. K. Alexander , K. C. Bible , et al. “2015 American Thyroid Association Management Guidelines for Adult Patients With Thyroid Nodules and Differentiated Thyroid Cancer: The American Thyroid Association Guidelines Task Force on Thyroid Nodules and Differentiated Thyroid Cancer,” Thyroid 26 (2016): 1–133, 10.1089/thy.2015.0020. 26462967 PMC4739132

[19] G. W. Randolph , Q. Y. Duh , K. S. Heller , et al., “The Prognostic Significance of Nodal Metastases From Papillary Thyroid Carcinoma can be Stratified Based on the Size and Number of Metastatic Lymph Nodes, as Well as the Presence of Extranodal Extension,” Thyroid 22, no. 11 (2012): 1144–1152, 10.1089/thy.2012.0043. 23083442

[20] H. Yamashita , S. Noguchi , N. Murakami , H. Kawamoto , and S. Watanabe , “Extracapsular Invasion of Lymph Node Metastasis is an Indicator of Distant Metastasis and Poor Prognosis in Patients With Thyroid Papillary Carcinoma,” Cancer: Interdisciplinary International Journal of the American Cancer Society 80, no. 12 (1997): 2268–2272, 10.1002/(SICI)1097-0142(19971215)80:12<2268::AID-CNCR8>3.0.CO;2-Q. 9404704

[21] I. Sugitani , A. Yanagisawa , A. Shimizu , M. Kato , and Y. Fujimoto , “Clinicopathologic and Immunohistochemical Studies of Papillary Thyroid Microcarcinoma Presenting With Cervical Lymphadenopathy,” World Journal of Surgery 22, no. 7 (1998): 731–737, 10.1007/s002689900461. 9606290

[22] M. H. Wu , W. T. Shen , J. Gosnell , and Q.‐Y. Duh , “Prognostic Significance of Extranodal Extension of Regional Lymph Node Metastasis in Papillary Thyroid Cancer,” Head & Neck 37, no. 9 (2015): 1336–1343, 10.1002/hed.23747. 24821456

