# Supplementary material for: Prognostic Impact of Internal Jugular Vein Invasion Through Extrathyroidal and Extranodal Extension in Papillary Thyroid Carcinoma
Source: World J Surg. 2026 Apr 9;50(5):1336–44. doi: 10.1002/wjs.70366 (PMC13206166; doi:10.1002/wjs.70366)
Supplement: Supplementary file 1 — Supporting Information S1 [file WJS-50-1336-s001.docx]

**SUPPLEMENTARY ONLINE CONTENT**

**eResults**

***Preoperative imaging and histopathological characteristics in cases with internal jugular vein (IJV) invasion***

Accurate preoperative assessment of IJV invasion is challenging, particularly in the absence of intraluminal tumor thrombus, an uncommon finding in papillary thyroid carcinoma. To further characterize preoperative imaging findings in cases requiring IJV resection, we retrospectively reviewed all preoperative CT images in the present series. Imaging findings were classified into four categories based on the relationship between the tumor and the IJV: complete luminal occlusion, obvious compression, contact with preserved luminal patency, and indeterminate findings.

Among the 17 cases with extrathyroidal extension (ETE) to the IJV, CT findings were categorized as follows: complete occlusion in 4 cases, obvious compression in 4 cases, contact with preserved luminal patency in 8 cases, and indeterminate findings in 1 case. Among the 47 cases with extranodal extension (ENE) to the IJV, CT findings were categorized as complete occlusion in 5 cases, obvious compression in 16 cases, contact with preserved luminal patency in 25 cases, and indeterminate findings in 1 case. Except for cases with complete luminal occlusion, preoperative imaging findings did not allow reliable differentiation of true IJV invasion.

The vast majority of cases were classified as classical PTC. A solid subtype was identified in 1 case in the ETE group and 1 case in the ENE group. No intraluminal tumor thrombus of the IJV was identified on pathological examination in any case.

***Patterns of recurrence and causes of death in patients with IJV invasion***

In the ETE group (n = 17), death occurred in 7 patients, including 6 deaths from thyroid cancer and 1 from other causes. Among patients who died of thyroid cancer, 1 had distant metastasis to the lung at the time of surgery, whereas the remaining 5 developed distant metastases during postoperative follow-up. The metastatic sites included the lung, the lung and brain, and bone in 3, 1, and 1 patients, respectively. In the ENE group (n = 47), 9 patients died during follow-up: 3 from thyroid cancer and 6 from other causes. Among patients who died of thyroid cancer, 1 had distant metastasis to the lung at the time of surgery, and the remaining 2 developed postoperative distant metastases to the lung.

After excluding patients who underwent R2 resection, cervical lymph node recurrence was observed in 3 of 13 patients in the ETE group and in 6 of 36 in the ENE group. No local recurrence at the primary resection site was observed in either group. Among patients without distant metastasis at initial diagnosis (cM0), 5 of 13 patients in the ETE group and 9 of 40 patients in the ENE group developed distant recurrence. In the ETE group, distant recurrence involved bone, lung, and both lung and brain in 1, 3, and 1 patients, respectively. In contrast, all distant recurrences in the ENE group consisted of lung metastases.
